# Selective inhibition of small-diameter axons using infrared light

**DOI:** 10.1038/s41598-017-03374-9

**Published:** 2017-06-12

**Authors:** Emilie H. Lothet, Kendrick M. Shaw, Hui Lu, Junqi Zhuo, Yves T. Wang, Shi Gu, Donna B. Stolz, E. Duco Jansen, Charles C. Horn, Hillel J. Chiel, Michael W. Jenkins

**Affiliations:** 1 Department of Pediatrics, Case Western Reserve University, Cleveland, OH USA; 2 Department of Biology, Case Western Reserve University, Cleveland, OH USA; 3 Department of Biomedical Engineering, Case Western Reserve University, Cleveland, OH USA; 4 Department of Cell Biology, University of Pittsburgh, Pittsburgh, PA USA; 5 Department of Biomedical Engineering, Vanderbilt University, Nashville, TN USA; 6 Biobehavioral Program in Oncology, University of Pittsburgh Cancer Institute, Pittsburgh, PA USA; 7 Department of Medicine: Division of Gastroenterology, Hepatology, and Nutrition, University of Pittsburgh School of Medicine, Pittsburgh, PA USA; 8 Department of Anesthesiology, University of Pittsburgh School of Medicine, Pittsburgh, PA USA; 9 Center for Neuroscience, University of Pittsburgh, Pittsburgh, PA USA; 10 Department of Neurosciences, Case Western Reserve University, Cleveland, OH USA

## Abstract

Novel clinical treatments to target peripheral nerves are being developed which primarily use electrical current. Recently, infrared (IR) light was shown to inhibit peripheral nerves with high spatial and temporal specificity. Here, for the first time, we demonstrate that IR can selectively and reversibly inhibit small-diameter axons at lower radiant exposures than large-diameter axons. We provide a mathematical rationale, and then demonstrate it experimentally in individual axons of identified neurons in the marine mollusk *Aplysia californica*, and in axons within the vagus nerve of a mammal, the musk shrew *Suncus murinus*. The ability to selectively, rapidly, and reversibly control small-diameter sensory fibers may have many applications, both for the analysis of physiology, and for treating diseases of the peripheral nervous system, such as chronic nausea, vomiting, pain, and hypertension. Moreover, the mathematical analysis of how IR affects the nerve could apply to other techniques for controlling peripheral nerve signaling.

## Introduction

Small-diameter axons play critical roles in sensory and motor systems. For example, small-diameter unmyelinated C-fibers carry nociceptive signals^1^, and small-diameter unmyelinated motor axons are often involved in control of peripheral glands and other autonomic structures^2^. If it were possible to selectively inhibit small-diameter axons, there would be many potential clinical applications. Electrical techniques for stimulation of the vagus nerve have already been found to have an effect on hypertension^3^, inflammation^4^ and obesity^5^. The existing techniques that modulate peripheral nerve signaling, however, do not selectively target small-diameter axons. Electrical inhibition (kilohertz high-frequency alternating current) blocks all neural activity^6^. Drugs that alleviate pain act systemically^7^. Optogenetics can target axonal sub-populations based on molecular markers^8^, but this technique requires genetic manipulations and may not be clinically applicable. Here, we report an alternative approach using IR light, which alters temperature due to tissue water absorption, to selectively, rapidly, and reversibly target small-diameter axons.

**Figure 1:**
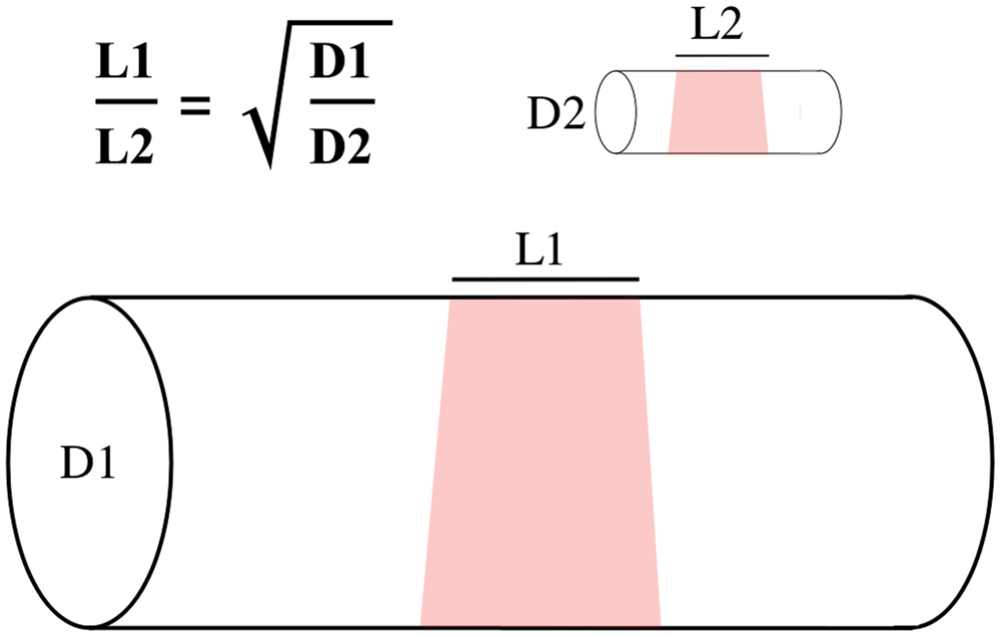
Schematic of the scaling of treatments applied along the surface of an axon. A mathematical analysis (see Supplement, Section 1) demonstrates that the equivalent length of a treatment applied along an axon’s surface scales as the ratio of the square root of the axon diameter. In the illustration shown, D1, diameter of the larger axon, is four times D2, the diameter of the smaller axon, and thus the equivalent effect on the large axon (L1) is twice as long as that needed to affect the smaller diameter axon (L2). This implies that less radiant exposure would be required to block the smaller-diameter axon than the larger-diameter axon.

Analysis of extracellular current application to peripheral nerves has demonstrated that larger-diameter axons are affected more than smaller-diameter axons, because current induced within the axon is proportional to axonal cross-section^9^. In contrast, if a modality acted primarily on ion channels on the axonal surface, a mathematical analysis of the cable equation demonstrates that its effects follow a different scaling law: rather than being proportional to cross-sectional area, the ratio of lengths scales as the square root of the ratio of the axon diameters [Fig. 1; see Supplement, Section 1]. A technology exploiting this approach might control small-diameter axons preferentially. Here, we demonstrate selective inhibition of small-diameter axons using IR light.

Previous work has shown that IR light can excite neurons^10^. Excitation using IR light has been demonstrated for cochlear implants, cortical stimulation, cardiac pacing, and the control of peripheral nerves^11–14^. Several mechanisms have been suggested for the excitatory effects of IR light: capacitive currents induced by thermal gradients^15^, actions on mitochondrial calcium currents^16, 17^, and actions on ion channels^18^.

More recently, IR light has been shown to inhibit neural and cardiac activity^19–22^. IR-induced inhibition may be due to an increase in baseline temperature, in contrast to IR-induced activation, which is believed to result from a brief (ms) spatiotemporal temperature gradient (dT/dt, dT/dz)^23^. By changing laser parameters (e.g., wavelength, pulse width, radiant exposure, repetition rate), one can produce brief temperature transients for stimulation or baseline temperature increases for inhibition. Laser-induced neural inhibition may result from non-uniform rate increases in temperature-dependent Hodgkin-Huxley gating mechanisms: the Na+ channel inactivation rate and K+ channel activation rate overwhelm the Na+ channel activation rate^24–27^. This theoretically causes a faster and weaker response, or complete but reversible block of action potential generation or propagation. IR light has many advantages for neural control including high spatial and temporal specificity, no electrical artifact or onset response, insensitivity to magnetic fields, and possibly different selectivity than electrical current.

**Figure 2:**
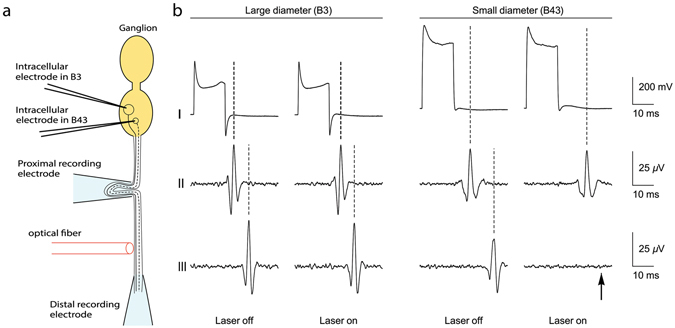
Selective block of an individual slower-conducting axon in *Aplysia californica*. (**a**) Experimental setup for selective optical inhibition. Two neurons, B3 and B43, were impaled and stimulated intracellularly. B3, a large-diameter cell, has a large-diameter axon, whereas B43, a small-diameter cell, has a small-diameter axon. Two suction recording electrodes were positioned along the length of the nerve, one proximal to the ganglion and one distal. The optical fiber (600 µm diameter) delivering the IR energy (1860 nm wavelength) was placed perpendicularly to the nerve between the recording electrodes. (**b**) Action potential recording from the large-diameter soma (B3) and axon and the small-diameter soma (B43) and axon. (I) Intracellular stimulation applied to the cell body. (II) Proximal recording. (III) Distal recording beyond the IR laser application. The B43 small-diameter axon was completely blocked at a radiant exposure of 0.106 J/cm^2^/pulse (arrow) whereas the B3 large-diameter axon remained unaffected.

To test whether smaller-diameter fibers would be preferentially inhibited by IR at the level of individual axons, we took advantage of an invertebrate preparation (*Aplysia californica*), in which prior studies showed that neurons with larger soma diameters typically have larger diameter axons and faster conduction velocities^28, 29^. We recorded from the somata of two identified neurons, B3 and B43, as shown in Fig. 2a. B3′s mean conduction velocity is 221% higher than that of B43 [p = 0.0271, Mann Whitney test; Figure S1a - box plot of conduction velocities for B3 versus B43]. We observed that lower radiant exposures (0.097 ± 0.026 J/cm^2^/pulse versus 0.126 ± 0.030 J/cm^2^/pulse) inhibited B43 compared to B3 [Fig. 2b; p = 0.0091, paired t-test; see Supplementary Figure S1b]; higher radiant exposures inhibited both axons [Supplementary Figure S2]. These effects were rapidly reversible (within 0.5 s).

**Figure 3:**
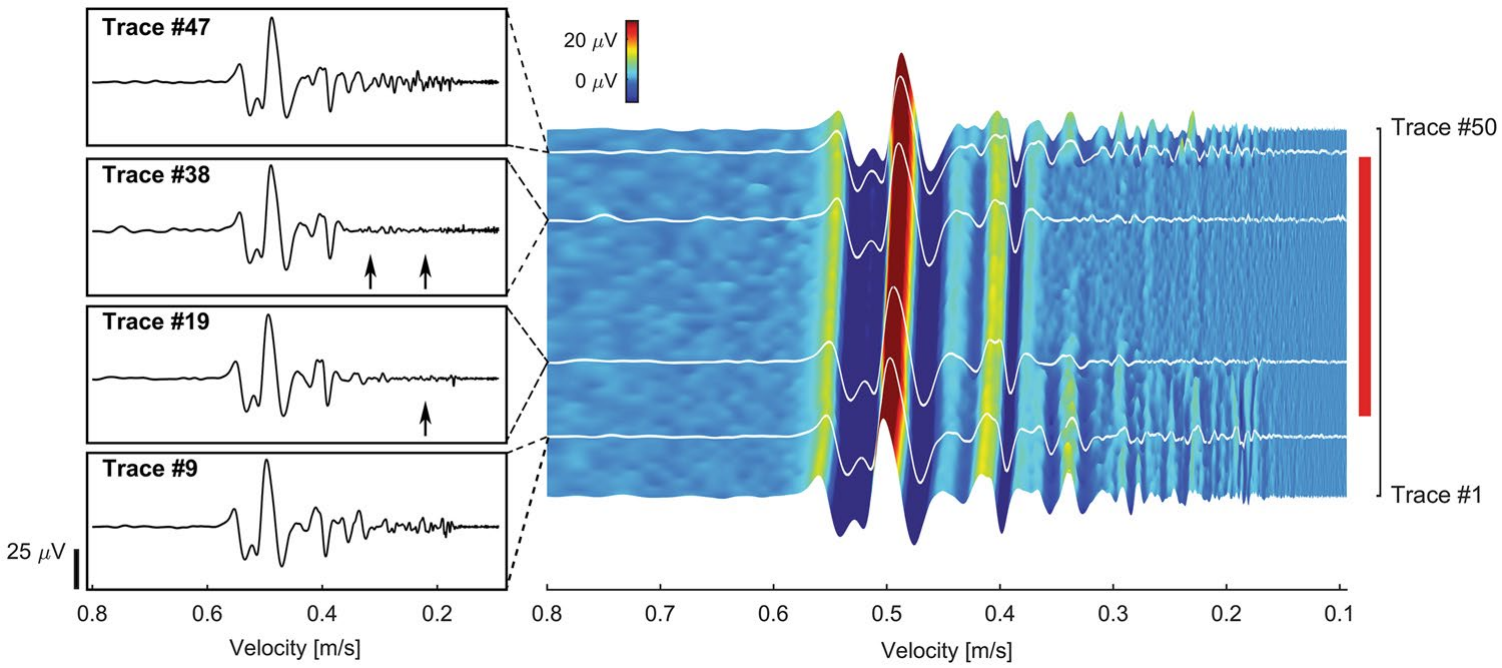
Selective block of slower-conducting CAP components in the *Aplysia californica* pleural-abdominal connective. (Left) Selected traces of CAP components corresponding to white lines on contour plot (right). (Trace 9) CAP before IR application. (Trace 19) CAP after IR application for 4.5 seconds. The slowest sub-populations (~0.2 m/s) are inhibited by IR light (arrow). (Trace 38) CAP after IR application for 14 seconds. Both the slowest (~0.3 m/s) and intermediate velocity populations (~0.4 m/s) are inhibited (arrows). (Trace 47) CAP after removal of IR light; all CAP components are present, indicating reversibility. (Right) Contour plot of CAP traces (electrical stimulation frequency, 2 Hz) illustrating progressive preferential block of slow components during IR application (red vertical bar; on, trace 11; off, trace 47). Conduction velocity (m/s) is plotted against trace number. A color bar denotes trace voltages. For analysis of data, see Figure S4.

To test whether populations of small-diameter unmyelinated fibers would be selectively inhibited by IR light, we used the pleural-abdominal connective of *Aplysia* [Figure S3 - setup], containing only unmyelinated axons whose most common axonal diameter ranges from 0.8–3 μm^30^. Electrical stimulation of the nerve generated a compound action potential (CAP), which included fast-conducting (large-diameter) and slow-conducting (small-diameter) axons. These components separate from one another over the length of the nerve. Within 11 seconds of the laser being turned on at a radiant exposure of 0.140 J/cm^2^/pulse, the slower components (0.43–0.18 m/s) of the CAP were blocked [Fig. 3, trace 38 compared to trace 9]. Once the laser was turned off, all components of the CAP returned [Fig. 3, trace 47]. Over the 50 traces, the process of inhibition selectively affected the slowest components [Fig. 3, contour plot]. To quantify the changes, we divided the CAP into regions at points of low variability [Figure S4a], and the rectified area under the curve (RAUC) was measured for each region [Figure S4b]. Experiments were conducted on 3 animals [data from a second preparation is shown in Figure S5]. Using chi-squared tests, slow-velocity components showed statistically significant reductions in RAUC when compared to the fast-velocity components in all three preparations. The average radiant exposure to block the smaller components was 0.110 ± 0.027 J/cm^2^/pulse, and the measured temperature increase was 9.7 ± 3.7 °C [Figure S6]. To demonstrate that the selective inhibition of axonal sub-populations is due to a thermal effect, we placed the *Aplysia* pleural-abdominal connective in a saline bath while controlling temperatures [Figure S7–setup]. As temperature increased, the slow-conducting components of the compound action potential were preferentially blocked [Figure S8, 25.7 °C]. As the bath temperature increased to still higher values, all components of the compound action potential eventually were inhibited [Figure S8, 40 °C].

**Figure 4:**
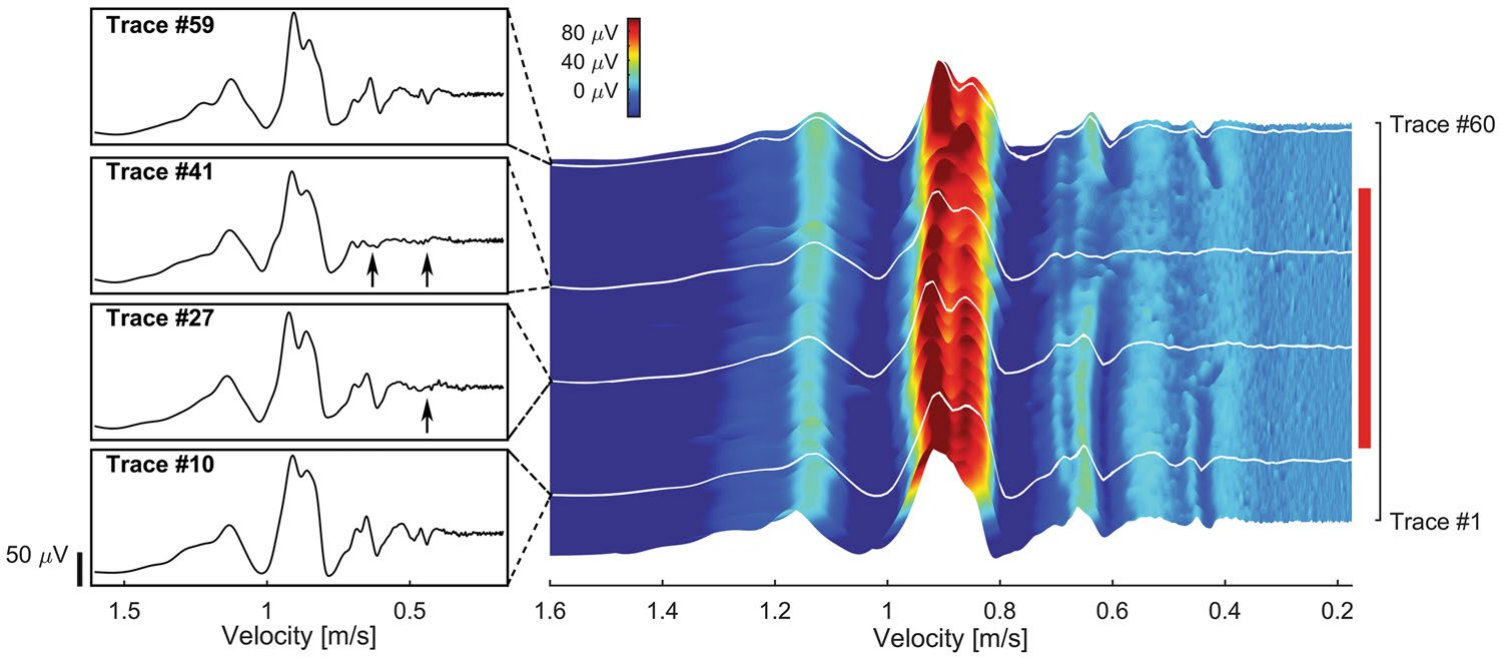
Selective block of slower-conducting CAP components in the *Suncus murinus* vagus nerve. (Left) Selected traces of vagal CAP corresponding to white lines on contour plot (right). (Trace 10) CAP before IR application. (Trace 27) CAP after IR application for 8.5 seconds. The slowest sub-population (~0.4 m/s) is inhibited (arrow). (Trace 41) CAP after IR application for 15.5 seconds. Both the slowest (~0.4 m/s) and intermediate velocity populations (~0.6 m/s) are inhibited (arrows). (Trace 59) CAP after removal of IR light; all CAP components are present, indicating reversibility. (Right) Contour plot of CAP traces (electrical stimulation frequency, 2 Hz) illustrating progressive preferential block of slow components during IR application (red vertical bar; on, trace 11; off, trace 51). Conduction velocity (m/s) is plotted against trace number. A color bar denotes trace voltages. For analysis of data, see Figure S8.

To test whether populations of small-diameter axons in vertebrates can be preferentially inhibited, even though they have different complements of ion channels than those in *Aplysia*, we studied the vagus of a mammal, the musk shrew *Suncus murinus*, a species used for emesis research on the vagus nerve because rats and mice lack an emetic reflex^31^. The vagus is a mixed nerve, containing both myelinated and unmyelinated axons. To measure changes in slow-conducting fibers, we reduced the fiber numbers by dissecting a small bundle of axons from the cervical end of the *in vitro* vagus preparation [Figure S9 – setup]. The CAP was induced by electrical shock at the upper thoracic end and was recorded from the cervical bundle. The laser was also applied to the cervical vagus between stimulating and recording electrodes. Within 14 seconds after the laser was turned on at a radiant exposure of 0.064 J/cm^2^/pulse, the slowest and intermediate components (0.68–0.35 m/s) of the CAP were blocked [Fig. 4 trace 41 compared to trace 10]. Once the laser was turned off, all components of the CAP returned [Fig. 4, trace 59]. Over the 60 traces, the process of inhibition selectively affected the slowest components [Fig. 4, contour plot]. To quantify the changes, we again divided the CAP into regions of low variability, and the RAUC was measured [Figure S10]. Each experiment was repeated 3 times/animal and in 3 different animals [data from a second preparation is shown in Figure S11]. Using Cochran-Mantel-Haenszel tests, slow-velocity components showed statistically significant reductions when compared to fast-velocity components in all preparations. The average radiant exposure to block the smaller components was 0.050 ± 0.012 J/cm^2^/pulse and the measured temperature increase was 2.9 ± 0.8 °C [Figure S12]. To demonstrate the presence of unmyelinated axons in the bundle, we performed transmission electron microscopy [Figure S13]. Unmyelinated axons ranged from 0.5–2.0 μm in feret diameter^32^, whereas myelinated axons ranged from 1.5–15.0 μm.

The experimental data strongly support the mathematical analysis, and thus suggest that any method for controlling axons that was applied primarily to the axonal surface would preferentially affect smaller-diameter axons. Thus, if a pharmacological agent (e.g., an ion channel blocker) was applied primarily to a length of the axonal surface, the analysis would predict that lower concentrations would be needed to block smaller-diameter axons than larger-diameter axons. Earlier studies suggested that axonal block due to heat may be due to the activation of voltage-dependent potassium channels overwhelming the currents through voltage-dependent sodium channels^24^, and thus any heating method that works via these ion channels might also be selective for block of smaller-diameter axons. Any optogenetic manipulations using light that primarily target ion channels on the surface of the axon should also show smaller-diameter specificity; this specificity should be tested in future studies. Since optogenetic manipulations use light to affect ion channels, applications of this technique to axons should also show smaller-diameter specificity. Finally, it is important to note that the mathematical principle is not limited by the specific limitations of the particular method used to modulate axonal activity.

Several lines of investigation are suggested by these results. Future studies will need to address the parameters of IR light that minimize radiant exposure, and determine safe levels of exposure both acutely and chronically, while still reliably targeting small-diameter axons. Since there is an initial rise in temperature when IR light is applied, it will be worthwhile exploring IR stimulation protocols that rapidly target a steady-state temperature that selectively inhibits small-diameter fibers. It is important to establish that the thermal load induced by IR does not produce either short- or long-term changes. At the lower radiant exposures used, temperature changes are similar to those observed under physiological conditions, so this may not be a limitation. We have focused primarily on unmyelinated axons, since these selectively carry sensory information such as pain^33^. Previous studies^26^ suggest that blocking myelinated fibers using IR might require multiple spots of light at multiple nodes of Ranvier, which could be explored in future studies. Since rapid pulses of IR have been used for neuronal excitation^14^, and may act along the surface of an axon^15^, it should also be possible to use IR to selectively excite small-diameter axons without exciting large-diameter axons. There may be other factors that are crucial for how axons respond to changes in temperature, such as TRP channels^34^ or involutions of the membrane^35^, and this could modulate the specificity of the IR technique.

The results presented in this study have broader implications for new technologies to control activity in the peripheral nervous system. Either alone, or in combination with other techniques, this approach could create novel methods for controlling, analyzing, and treating diseases of the nervous system.

## Methods

### Animal models

#### Aplysia model

The marine mollusks *Aplysia californica* (Marinus Scientific, Long Beach, CA) were maintained in an aerated aquarium circulating artificial seawater (Instant Ocean, Aquarium Systems, Mentor, OH) kept at 16–17 °C, with a 12-hour dark/12-hour light cycle. The aquarium contained macroalgae and organisms simulating the ecosystem in which marine slugs normally live. The animals were fed a diet of dried seaweed every other day. Inter-bite intervals (3–5 s) in response to a seaweed strip indicating normal health were used for selecting animals.

### Shrew model and ethics statement

Experimentally naïve adult male musk shrews were used (*Suncus murinus*; >35 days of age). The male shrew was used as they were often larger in size than females and generally had larger and thicker vagus nerves, making the surgeries and bundle dissections easier and more likely to succeed. Musk shrews were obtained from a breeding colony at the University of Pittsburgh Cancer Institute and were descendants from animals acquired from the Chinese University of Hong Kong, a Taiwanese strain. Animals were singly housed in clear plastic cages, with a filtered air supply, under a 12-hour standard light cycle (lights on at 07:00 AM), in a temperature (~23 °C) and humidity (~40%) controlled environment. Food and drinking water were freely available, but food was removed 2 hours before euthanasia and removal of the vagus nerve. Food consisted of a mixture of 75% Purina Cat Chow Complete Formula and 25% Complete Gro-Fur mink food pellets. Experiments were approved by the University of Pittsburgh Institutional Animal Care and Use Committee and conducted in compliance with USDA guidelines. Animals were housed in an Association for Assessment and Accreditation of Laboratory Animal Care international-accredited animal care facility.

### Laser

For all inhibition experiments, a tunable diode laser (Capella; Lockheed-Martin-Aculight, Bothell, WA) with a wavelength λ = 1860 nm was used. Block was induced by applying 200 µs pulses at 200 Hz. The IR laser was coupled into an optical fiber whose diameter corresponded to the cross-section of the target nerve. For all *Aplysia* experiments, the diode laser was coupled to a 600 µm multimode optical fiber (P600-2-VIS-NIR, Ocean Optics, Dunedin, FL) positioned at a 90° angle over the nerve using a micromanipulator. The optical fiber gently touched the nerve sheath. Shrew experiments were similar to those in *Aplysia*, except that a 400 µm optical fiber was used.

At the end of each experiment, the pulse energies at which block was obtained were measured using a pyroelectric energy meter (PE50BB, Ophir-Spiricon, North Logan, UT). From these measurements, the radiant exposure (J/cm^2^/pulse) effective at producing optical block could be established by dividing the individual pulse energies by the laser spot size. Instead of making assumptions to determine the laser spot size at the axons, we report the radiant exposures at the fiber tip.

We used a thermal camera (FLIR A325sc, Wilsonville, OR) along with the ResearchIR software to assess laser-induced temperature changes to the tissue as reported in our previous publication^22^. Preliminary tests comparing temperature rise in nerves in Krebs solution and water alone showed no discernible differences so we used water to simplify the experiments. Briefly, we cut one rounded edge of a Petri dish off and replaced it with a flat cover slip and filled it with water. We then positioned a 400 µm (shrew experiments) or 600 µm (*Aplysia* experiments) optical fiber just barely touching the surface of the water and with the cross-section bisected by the glass-water interface. By assuming an axially symmetric temperature distribution and taking into account that glass has a high thermal conductivity and a low specific heat compared to water, thermal imaging at the cover slip surface provided an accurate measure of temperature distribution in depth through the middle of the heated region. We tested a range of laser energies that corresponded to values used in our experiments. For each laser level, we recorded for 700 seconds. The laser was applied for a 300-second window between 101–400 seconds, which provided time for the temperature to reach a steady state and return to baseline after turning the laser off. Plots in Figures S6 and S12 show the temperature versus time at the depth where the center of the nerve would have been for the *Aplysia* and shrew, respectively. To determine the actual temperature threshold for inhibition within the nerve, the time point on the temperature profile for a specific radiant exposure corresponding to how long it took to achieve block was used. We employed a piecewise cubic Hermite interpolating polynomial (PCHIP) interpolation when the measured radiant exposure fell between the measured traces.

### Experiments

#### Intracellular identified cell and axon experiments

*Aplysia californica* (a total of N = 7 animals, 8 nerves) weighing 250–350 g were used for these experiments. Animals were anesthetized with an injection of MgCl_2_ (~50% of body weight) prior to dissection. Once anesthetized, the buccal ganglion and associated nerve, buccal nerve 2 (BN2), were dissected out of the animal. The nerve was cut distally prior to the trifurcation into separate branches. After pinning the buccal ganglion to the dish containing Sylgard (Dow Corning, Auburn, MI), the protective sheath of the buccal ganglion was removed to allow access to the nerve cell somata with intracellular glass electrodes. The nerve and the ganglion were immersed in a mixture of high-divalent cation *Aplysia* saline (270 mM NaCl, 6 mM KCl, 120 mM MgCl_2_, 33 mM MgSO_4_, 30 mM CaCl_2_, 10 mM glucose, and 10 mM 3-(N-morpholino) propanesulfonic acid, pH 7.5).

Intracellular glass electrodes were used to impale identified neurons B3 and B43 to record and control their voltage [Fig. 2a]. The electrodes were pulled from thin-walled filament capillary glass (1.0 mm outer diameter, 0.75 mm inner diameter, A-M Systems) using a Flaming/Brown micropipette puller (model P-80/PC, Sutter Instruments, Novato, CA) and had an inner diameter ranging from 3–6 µm. Electrodes were backfilled with 3 M potassium acetate before use. The bridge was balanced for stimulation and recording. The identified cells were stimulated at a frequency of 2 Hz. Intracellular signals were amplified using a DC-coupled amplifier (model 1600, A-M Systems).

To record action potentials travelling down the length of the nerve, extracellular suction electrodes were positioned along the length of BN2. The electrodes were made by pulling polyethylene tubing (Becton Dickinson, #427421; outer diameter 1.27 mm, inner diameter 0.86 mm) placed over a flame to obtain an electrode whose diameter matched the nerve. Prior to suctioning the nerve, each extracellular electrode was filled with high-divalent cation *Aplysia* saline. Two extracellular electrodes were placed on BN2: one *en passant* electrode mid-way along the length of the nerve, and one suction electrode at the cut end of the nerve. An Ag/AgCl-coated wire was inserted in the recording electrodes. Recordings from extracellular electrodes were amplified using an AC-coupled differential amplifier (model 1700, A-M Systems, Sequoia, WA) and filtered using a 500 Hz low-pass and a 300 Hz high pass filter. Data were digitized and recorded for analysis using AxoGraph X.

Thresholds for reliably inducing action potentials were determined individually for the larger-diameter neuron (B3) and axon, and the smaller-diameter neuron (B43) and axon. Conduction velocities were determined for each neuron and axon (N = 6 for B3, N = 3 for B43). Radiant exposure block thresholds were then established for the small-diameter B43 axon and the large-diameter B3 axon (N = 5 for pairs of axons). The neurons were electrically stimulated after infrared light application to assess nerve health and IR block reversibility.

#### *Aplysia* whole nerve *in vitro* experiments

To separate the axonal sub-populations with different conduction velocities, we chose to use a longer nerve (the *Aplysia* pleural-abdominal connective). Larger animals weighing 350–410 g were used because they have longer nerves (N = 7 animals). The ganglia on either end of the nerve were dissected away. The nerve was placed in a Sylgard recording dish containing *Aplysia* saline (460 mM NaCl, 10 mM KCl, 22 mM MgCl_2_, 33 mM MgSO_4_, 10 mM CaCl_2_, 10 mM glucose, and 10 mM 3-(N-morpholino) propanesulfonic acid, pH 7.5), and its sheath was pinned down.

To stimulate the nerve, a monopolar extracellular suction electrode was placed at one cut end of the nerve [Figure S3, left]. The stimulation electrode was grounded using a return electrode placed in the dish’s saline. The nerve was stimulated at a frequency of 2 Hz. A bipolar extracellular recording electrode, composed of an *en passant* and a suction electrode, was placed at the other end of the nerve [Figure S3, right]. The bipolar recording electrode reduces recording noise. Electrodes were filled with *Aplysia* saline before suctioning the nerve to preserve its viability. Signals were amplified using the extracellular amplifier described above, and the nerve CAP was digitized and recorded using AxoGraph X.

Thresholds for reliably inducing all CAP components were determined. We observed that if currents significantly higher than threshold were used, we sometimes recruited additional components to the CAP that were of intermediate velocity and highly resistant to thermal block. To prevent this from happening, we ensured that stimulation amplitudes were just above threshold. Conduction velocities were determined for the different CAP sub-components (N = 3). Radiant exposure block thresholds were then established for the slower, smaller-diameter sub-components (N = 7). The nerve was electrically stimulated after infrared light application to assess nerve health and IR block reversibility.

The *in vitro* bath heating experiments (N = 4) used a similar preparation to the one described above. A stimulation suction electrode was placed on one end of the nerve and a monopolar recording suction electrode was placed on the other end of the nerve [Figure S7]. The nerve was stimulated at a frequency of 2 Hz, and the signal was amplified using an external amplifier.

Current amplitude threshold for reliable stimulation of all CAP components was determined at room temperature (21.5–24.5 °C). *Aplysia* saline, warmed using a water bath (model EX-211, Neslab) and an in-line heating system (model TC-344C [temperature controller], SH-27B [in-line heater], Warner Instruments), was perfused using a peristaltic pump (model MasterFlex 7524–10, Cole Parmer) through the dish. Its temperature was monitored using a temperature probe (model SDL200, Extech) and digitized. The bath temperatures tested ranged from room temperature to 39.8 ± 0.4 °C. After reaching 39.8 ± 0.4 °C, cold saline was added to the bath to return it to room temperature and assess the nerve’s health. The nerve was continuously stimulated throughout the experiment to monitor its ability to conduct at the varying temperatures.

#### Shrew whole nerve *in vitro* experiments

Animals (N = 3 nerves from 3 separate animals) were euthanized by exposure to CO_2_ until lack of respiration, followed by cervical dislocation. The thoracic cavity was opened to reveal the heart and an incision was made in the cardiac apex to drain the blood. This method was used to reduce bleeding in the neck when excising the vagus. A midline incision was then made in the ventral surface of the neck, including a cut through the clavicle bones to expose the left vagus trunk, which exposed the segment of the vagus from above the heart to the nodose ganglion. This cervical plus thoracic vagal segment was removed and placed in cold Krebs solution (5 to 7 °C). The time from euthanasia to placing the nerve in Krebs solution was less than 5 min. The nerve was then further dissected in a dish containing Krebs (which was continually oxygenated) to remove excess connective tissue before placement in a three-compartment chamber for electrophysiology recordings [Figure S9]. Krebs solution was perfused through the middle compartment at a rate of 5.1 ml/min and the temperature was controlled to be 37 °C. The laser was applied just outside the middle chamber, and thus the temperature at the site of laser application was close to body temperature.

In the nerve stimulation compartment, the nerve was pinned at the end and draped across two platinum-iridium hook electrodes (separated by 0.5 mm). The nerve and electrodes were encased in Kwik-cast silicone (WPI, Sarasota, FL) and the compartment was filled with mineral oil. Nerve stimulation was produced by applying biphasic pulses through the stimulation electrodes (0.5 ms duration; 0.5 s inter-pulse interval; 0.04 to 0.11 mA, depending on which current level would allow for reliable stimulation of all axonal sub-populations. Once selected, the current level was kept constant throughout the experiment).

The recording compartment was also filled with mineral oil, and the nerve was positioned across a reference electrode. When recording from the whole vagus, the noise obscured the activity of slower-conducting fibers. For that reason, we dissected out small bundles from the cervical vagus from which to record. In each experiment, a nerve bundle was dissected from the nerve trunk and wrapped around a recording electrode. Signals were acquired at an amplification of 5,000 using a differential AC amplifier (P511, Grass Instruments, Natus Medical Inc, Pleasanton, CA; 100 and 1,000 Hz cutoffs) and recorded to computer (Spike 2, CED, Cambridge, England).

The experimental design of the shrew *in vitro* experiments closely followed the experimental design used for the *Aplysia* whole nerve *in vitro* experiments (see above). The only difference is that each experiment was repeated three times in each animal.

For transmission electron microscopy (TEM), nerves were harvested and immersion fixed (2.5% glutaraldehyde, 2% paraformaldehyde in PBS) overnight at 4 °C. Following fixation, tissue was washed 3x in PBS then post-fixed in aqueous 1% OsO_4_, 1% K_3_Fe(CN)_6_ for 1 hour. Following 3 PBS washes, the tissue was dehydrated through a graded series of 30–100% ethanol, 100% propylene oxide, and then infiltrated in a 1:1 mixture of propylene oxide: Polybed 812 epoxy resin (Polysciences, Warrington, PA) for 1 hour. After several changes of 100% resin over 24 hours, the tissue was embedded in molds, cured at 37 °C overnight, followed by an additional hardening at 65 °C for two more days. Semi-thin (300 nm) cross-sections were heated onto glass slides, stained with 1% toluidine blue and imaged using light microscopy. Ultrathin (60 nm) cross-sections of nerve were collected on copper grids, stained with 1% uranyl acetate for 10 minutes, followed by 1% lead citrate for 7 minutes. Sections were imaged using a JEOL JEM 1400 transmission electron microscope (Peabody, MA) at 80 kV fitted with a side-mount AMT 2 k digital camera (Advanced Microscopy Techniques, Danvers, MA).

### Data and Statistical Analysis

For single cell experiments, a Mann Whitney test determined whether conduction velocities in axons projecting from B3 and B43 were statistically different. A paired t-test determined whether threshold radiant exposure levels for inhibiting action potentials in B3 and B43 were statistically different.

For whole nerve experiments, data were digitally filtered using a 50 Hz 4^th^ order high-pass Butterworth filter, and a 1000 Hz 4^th^ order low-pass Butterworth filter. By identifying the onset of the artifact, each stimulation trial was extracted. Because waveform shapes can be changed both by shift in the underlying action potentials with temperature as well as by complete block^36^, stable regions within the CAP were identified by finding the areas of lowest variance across all stimulation traces. Within each of these stable regions, corresponding to different conduction velocities, the rectified area under the curve (RAUC) was calculated. Medians were plotted, surrounded by dashed lines representing the first and third quartiles of the data for successive stimulation groups. Results were converted to binary categorical data (1 - no significant decrease of RAUC, 0 - RAUC was reduced to less than 1/e compared to traces recorded before the IR laser was on). The same experiment was repeated three times on three different preparations, and the results were analyzed using the Cochran-Mantel-Haenszel test to remove any possible influences from biological variability among the three experiments. For the *Aplysia* data, the standard chi-squared test was used. When multiple comparisons were tested in the same experimental set, the Bonferroni correction was applied to control the overall Type I error. To reach statistical significance, the overall *p* value was set at 0.05 before the Bonferroni correction.

## Electronic supplementary material

Supplemental material
