# Supplementary material for: Selective inhibition of small-diameter axons using infrared light
Source: Sci Rep. 2017 Jun 12;7:3275. doi: 10.1038/s41598-017-03374-9 (PMC5468240; doi:10.1038/s41598-017-03374-9)
Supplement: Supplementary file 1 — Supplemental material [file 41598_2017_3374_MOESM1_ESM.doc]

**Selective inhibition of small-diameter axons using infrared light**

Emilie H. Lothet^1,2^, Kendrick M. Shaw^2^, Hui Lu^2^, Junqi Zhuo^1,3^, Yves T. Wang^1^, Shi Gu^3^, Donna B. Stolz^4^, E. Duco Jansen^5^, Charles C. Horn^6,7,8,9^, Hillel J. Chiel^2,3,10^, Michael W. Jenkins^1,3,*^

^1^Department of Pediatrics, Case Western Reserve University, Cleveland, OH

^2^Department of Biology, Case Western Reserve University, Cleveland, OH

^3^Department of Biomedical Engineering, Case Western Reserve University, Cleveland, OH

^4^Department of Cell Biology, University of Pittsburgh, Pittsburgh, PA

^5^Department of Biomedical Engineering, Vanderbilt University, Nashville, TN

^6^Biobehavioral Program in Oncology, University of Pittsburgh Cancer Institute, Pittsburgh, PA

^7^Department of Medicine: Division of Gastroenterology, Hepatology, and Nutrition, University of Pittsburgh School of Medicine, Pittsburgh, PA

^8^Department of Anesthesiology, University of Pittsburgh School of Medicine, Pittsburgh, PA

^9^Center for Neuroscience, University of Pittsburgh, Pittsburgh, PA

^10^Department of Neurosciences, Case Western Reserve University, Cleveland, OH

^*^Correspondence to mwj5@case.edu

**Section 1. Diameter scaling of effects along the length of axons**

Here we prove that the minimum thermal block width scales with the square root of axonal diameter. We will do this by proving a more general theorem – that for any two axons of different diameter, if their physical properties along the length of the axon are identical when position is scaled by the square root of diameter, then their behavior will be identical in this scaled coordinate system. To do this, we will derive a somewhat more general version of the cable equation, which does not assume that the electrical properties of the cable, e.g., capacitance and conductance, are constant in space and time. We then make the dependence on diameter explicit. Finally, we rewrite this equation in a new coordinate system (where length is scaled by the square root of diameter), and show that diameter disappears from the equation in the new coordinate system.

We first define some of the constants and variables we will be using. Assume the axon is a cylinder of fixed diameter *b*. Let
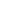
 be the membrane capacitance per unit area (which may vary over space or time). Then the capacitance per unit length along the axon,
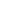
, is


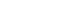
 (1)

Similarly, let
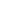
 be the conductance per unit area of the *k*-th current. The conductance per length of the current is then


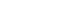
 (2)

Let
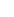
 be the resistivity of the axoplasm; then the resistivity per unit length is


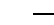
 (3)

We now derive a more general form of the standard cable equation that does not assume that the capacitance, conductance, and resistivity are fixed, so that they may vary over time and space. We first use Ohm’s law to write the voltage drop along the axon:


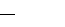
 (4)

where
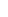
 is the position along the length of the axon and
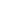
 is the current flowing along the axon in the direction of increasing
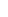
. We then use conservation of charge to note that the change in current along the axon is equal to the net current flowing into the axon, or


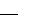
 (5)

where
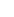
 is the net current flowing across the membrane per unit length.

To combine these two equations, we first differentiate (4), yielding


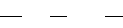
 (6)

we now use (4) to substitute for
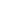
, producing


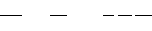
 (7)

Solving for
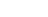
, we have


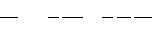
 (8)

Combining this with equation (5) gives us


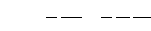
 (9)

We now write out the components of the membrane current. We use the definition of capacitance to write the charge per unit length stored on the membrane as


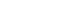
 (10)

where *V* is the membrane potential at the given axial slice of the axon. We then differentiate
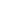
 to get the capacitive current per unit length flowing into the axon,


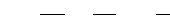
 (11)

where *t* is time. Note that the negative sign was introduced to preserve the direction of the current so that charging the membrane capacitance with a positive potential generates an outward (negative) flow. We can use Ohm’s law to write the current per unit length flowing into the axon through channel *k*,


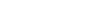
 (12)

where
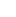
 is the reversal potential of channel *k*. The total membrane current is just the sum of the capacitive and channel currents, or


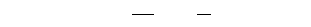
 (13)

Combining this equation with (9) and multiplying by –1 yields our desired more general version of the standard cable equation:


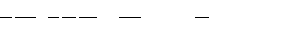
 (14)

We now attempt to understand the effects of diameter in our equation. If we assume that the axon diameter only enters the equation through its effects on
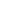
,
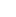
, and
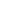
, we can use their definitions (equations 1-3) to make the dependence on diameter explicit in our cable equation (14),


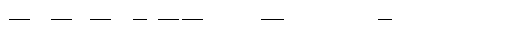
 (15)

Dividing through by
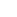
 and grouping *b* with the partial derivatives, we can rewrite this as


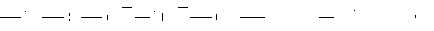
 (16)

We now perform a change of coordinates in an attempt to isolate the effects of the diameter *b*. Let us define a new spatial coordinate *u* such that


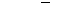
 (17)

Then


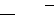
 (18)

and thus for an arbitrary smooth function *f*,


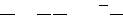
 (19)

and


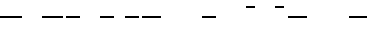
 (20)

Applying these identities to (16), we have


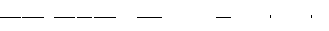
 (21)

This final version of the cable equation, (21), proves our hypothesis. To see this, note that it has no dependence on the diameter *b*, only *u*. Thus, if two axons have the same membrane capacitance, conductance per unit area and axonal resistivity when expressed in *u*-coordinates, then they have identical behavior in *u*-coordinates. As a specific example, if an axon with diameter *b* can be blocked by a high-temperature region of a minimum length *l*, then an axon of diameter *c* with otherwise identical properties will be blocked by a high-temperature region of minimum length
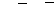
. The power and generality of this mathematical model allow us to assert that any particular biophysical model of an axon that satisfies the assumptions of the cable equation will show the same scaling law. Since the model is completely general, it makes predictions about any modality that affects neurons along their surface.

**Section 2. Inhibition at the level of single axons in *Aplysia***


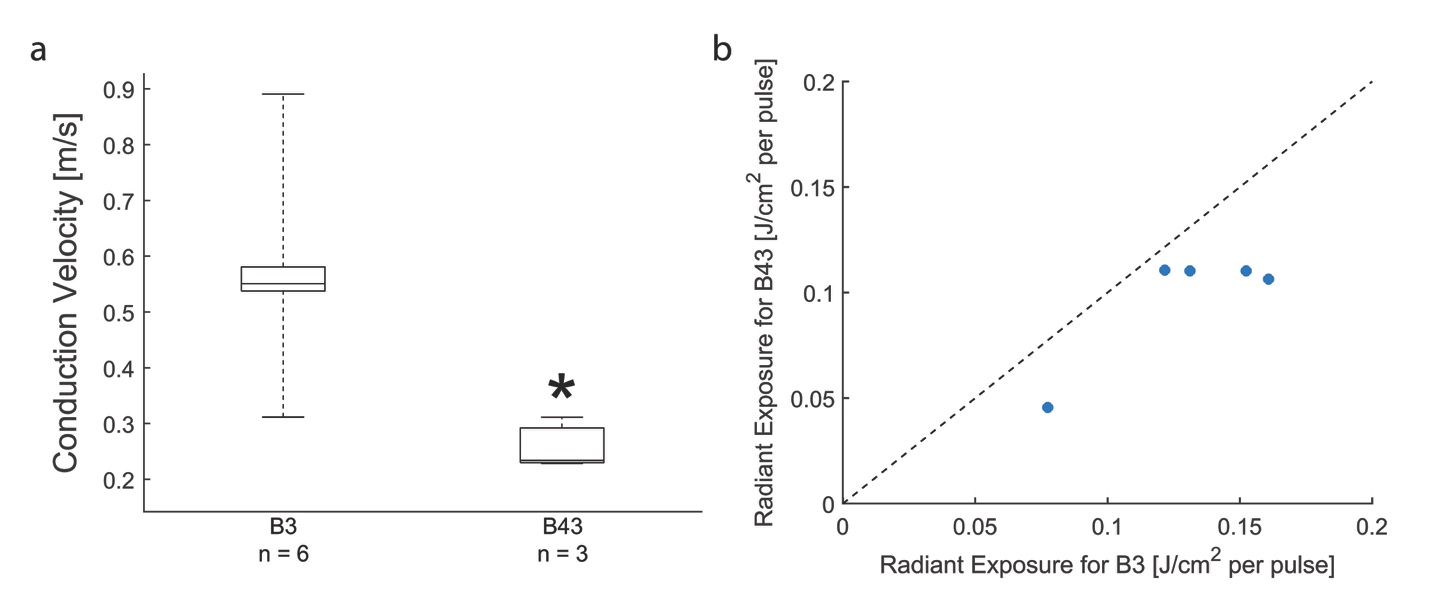


**Figure S1: Larger diameter neurons have higher conduction velocity axons and require higher radiant exposure to be blocked. (a)** Box and whisker plots of conduction velocities for neuron B3 (larger diameter) and neuron B43 (smaller diameter). The conduction velocity for B43 is significantly lower than that for B3 [B3: 0.57±0.19 m/s (N = 6), B43: 0.26±0.05 m/s (N = 3); p = 0.0271, Mann Whitney test) **(b)** Comparison of thresholds of radiant exposure for B3 versus B43 (matched pairs; N = 5 animals). If the radiant exposures were identical, data points would lie on the dashed line. Instead, the exposures needed to block B43 (0.097±0.026 J/cm^2^/pulse) are lower than those needed to block B3 (0.126±0.030 J/cm^2^/pulse), and lie below the line (p = 0.0091, paired t-test).


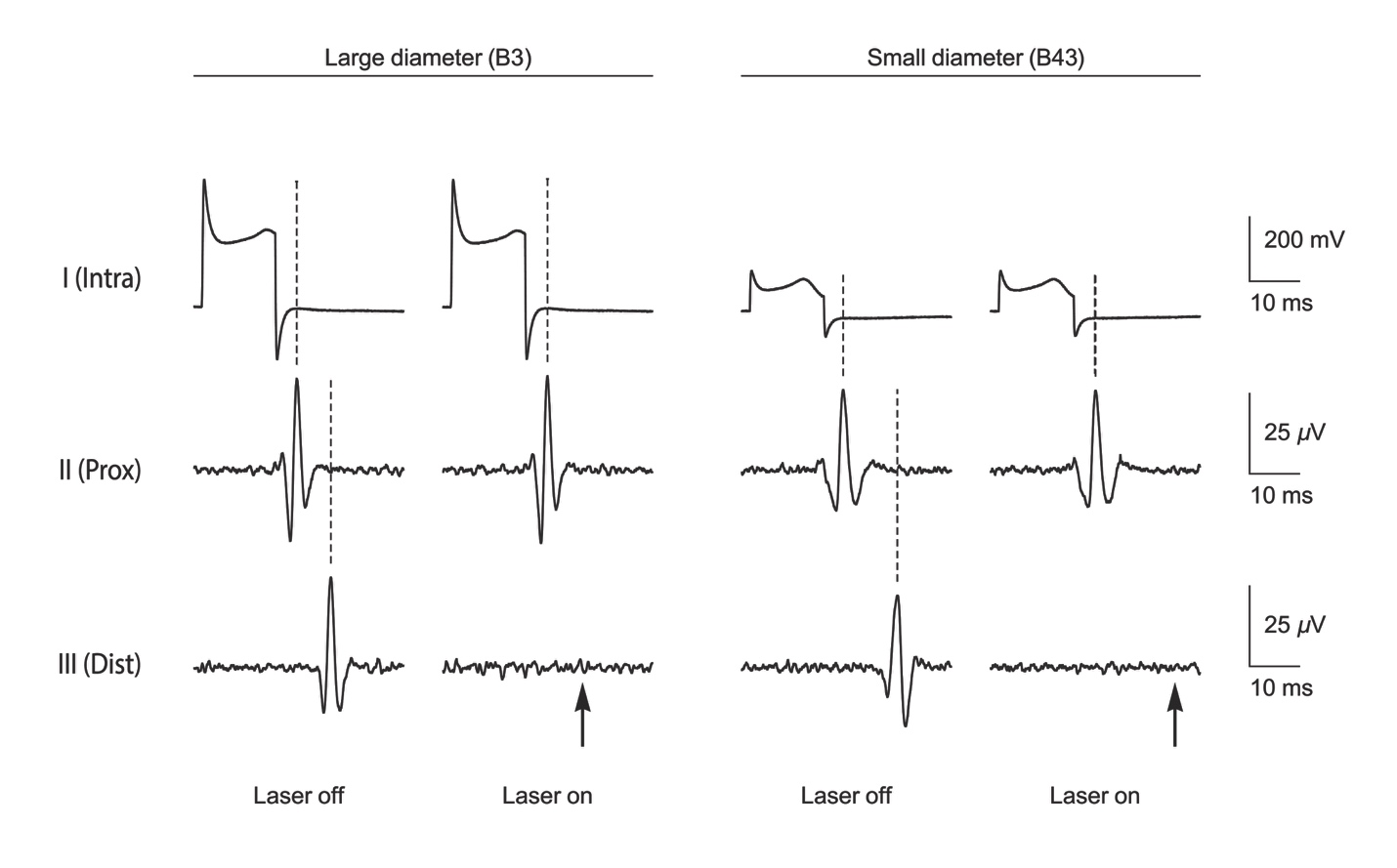


**Figure S2: Block of both slow- and fast-conducting axons in *Aplysia californica*.** Action potential recordings from the large-diameter axon (B3) and the small-diameter axon (B43). **(I)** Intracellular stimulation applied to the nerve cell bodies. **(II)** Recording obtained at the proximal recording electrode. **(III)** Recording obtained at the distal recording electrode, positioned beyond the optical fiber. Using a higher radiant exposure (0.161 J/cm^2^/pulse) than used in Figure 2 (0.106 J/cm^2^/pulse, in which only the small-diameter axon was blocked), both B3 (large-diameter axon) and B43 (small-diameter axon) were completely blocked by application of IR light (arrows).

**Section 3. Inhibition of small, slow-conducting fibers in whole nerves of *Aplysia***


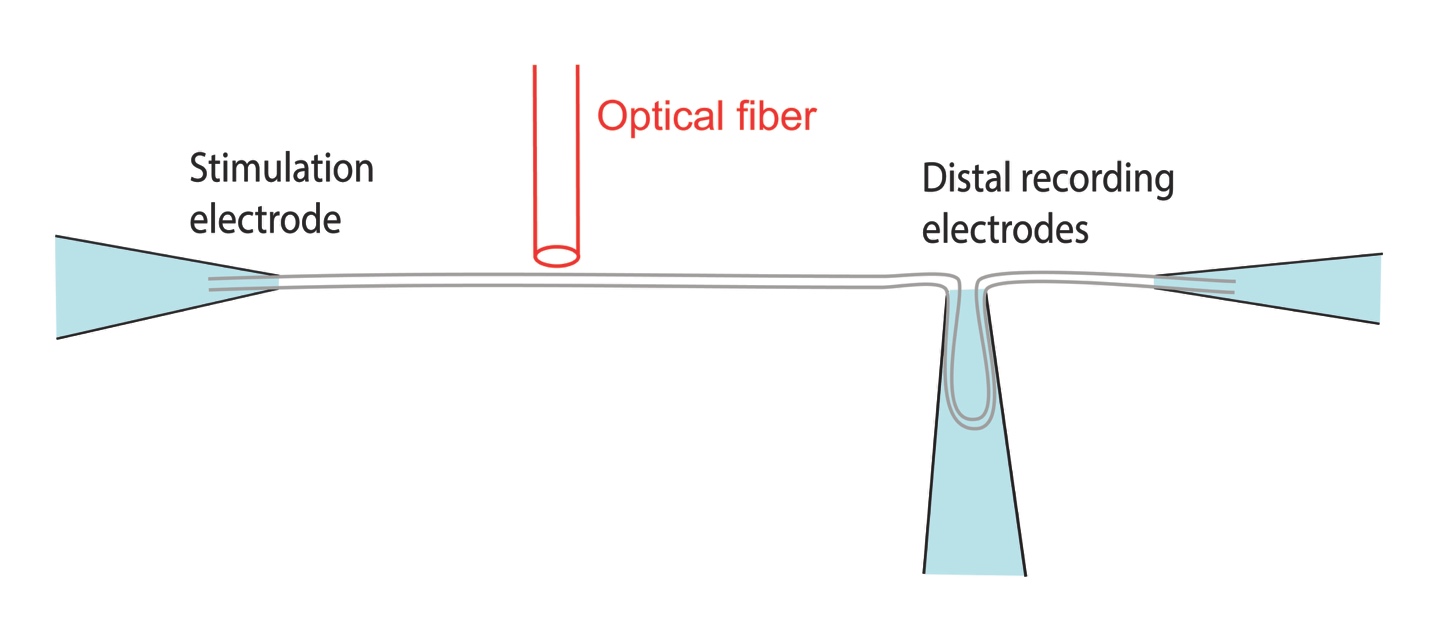
**Figure S3: Experimental setup for optical inhibition in the pleural-abdominal connective of *Aplysia californica*.** A stimulation suction electrode was placed on one end of the nerve (left) and a bipolar recording suction electrode was placed on the other end of the nerve (right). The IR optical fiber (600 µm diameter, 1860 nm wavelength) was positioned perpendicularly to the nerve between the stimulation and recording electrodes.


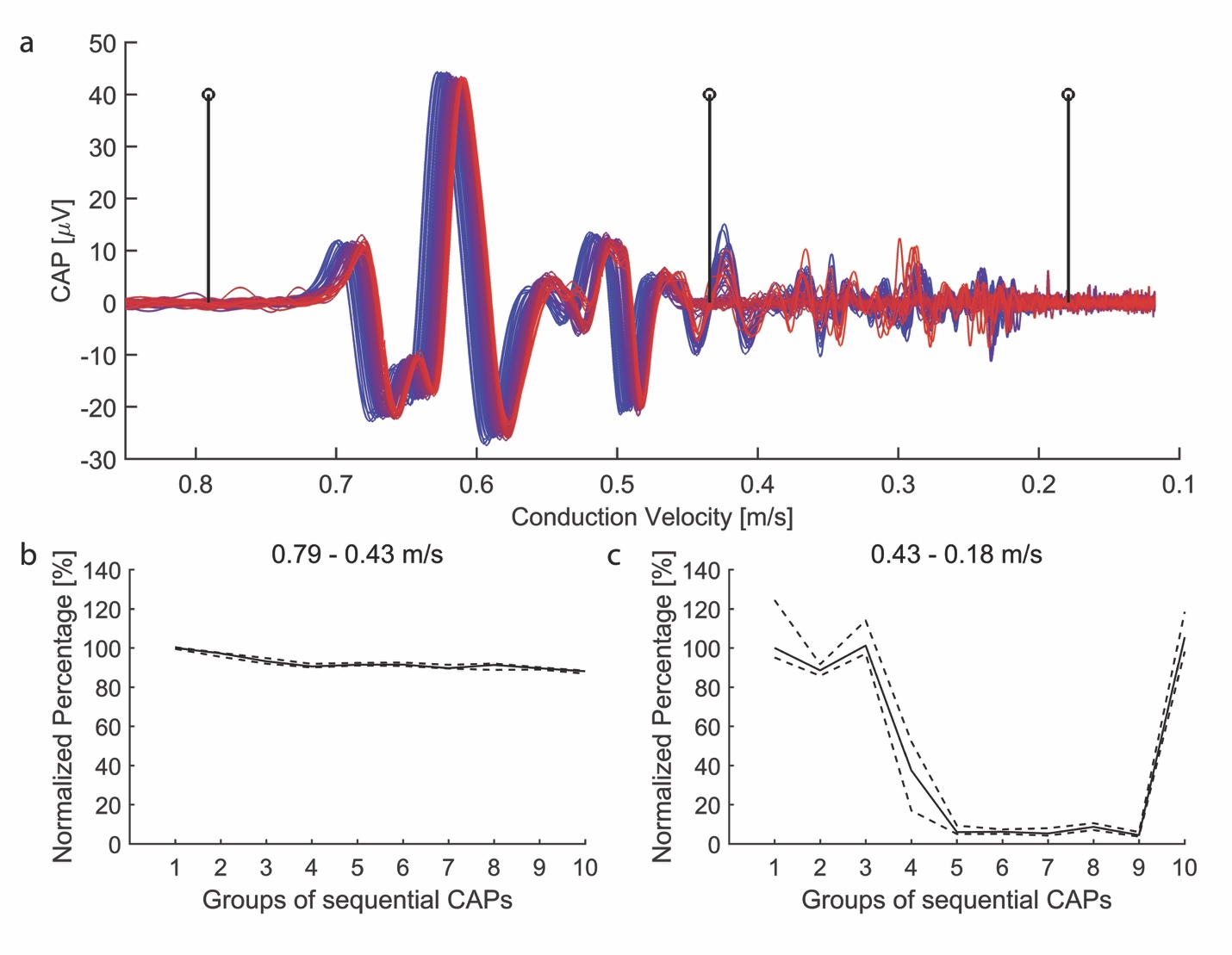


**Figure S4: Determining stable regions and quantifying change of the rectified area under the curve (RAUC) for CAP components in *Aplysia californica*** **(a)** Superposition of 50 CAP traces during the experiment shown in Figure 3 (colors change from blue to red during the sequence of CAPs). Note the regions of low variability, marked using bold black lines marked with circles. These were used to divide the data into regions of different conduction velocity ranges. **(b)** Plots of medians (dark line) surrounded by the first quartile (upper dashed line) and third quartile (lower dashed lines) for successive groups of 4 recordings in the faster velocity (conduction velocity range: 0.79 – 0.43 m/s) region of the CAP. These results quantify the data shown in that velocity range for Figure 3. Little change is observed in the median values even during the application of the IR laser light. **(c)** Plots of medians (dark line) surrounded by the first quartile (upper dashed line) and third quartile (lower dashed lines) for successive groups of 4 recordings in the slower velocity (conduction velocity range: 0.43 - 0.18 m/s) region of the CAP. These results quantify the data shown in that velocity range for Figure 3. Note the large decrease in the median amplitude during the application of the IR laser light. Using a chi-squared test, slow-velocity components (0.43 - 0.18 m/s) showed statistically significant reductions in RAUC when compared to the fast-velocity (0.79 - 0.43 m/s) components.

**
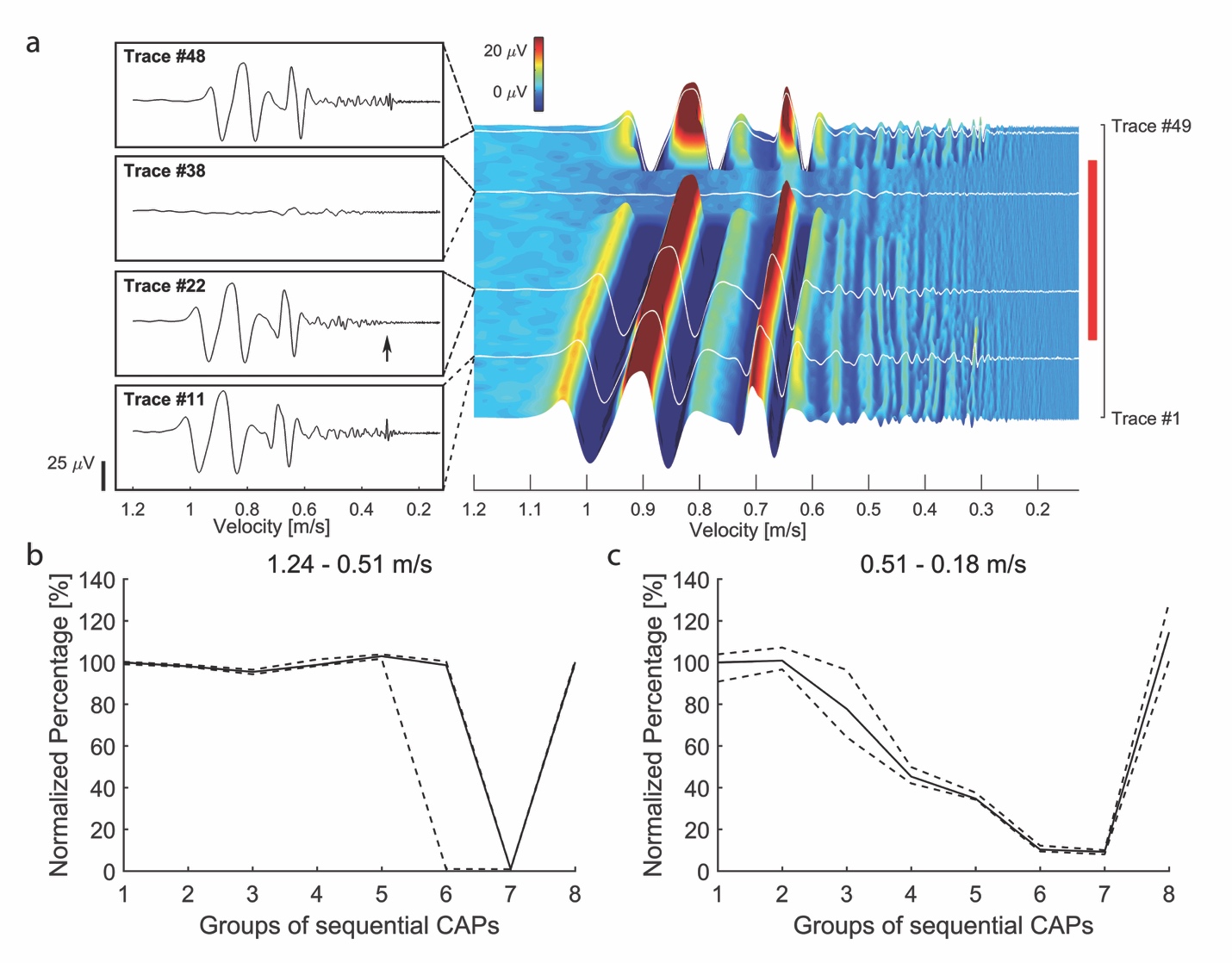
**

**Figure S5:** **Sequential block of all axonal sub-populations in *Aplysia californica* compound action potentials (a - Left)** Slower-conducting components of the CAP are blocked earlier as IR light is applied. Selected traces of CAP components corresponding to white lines on contour plot (right). **(Trace 11)** CAP before IR application. **(Trace 22)** CAP after IR application for 5 seconds. The slowest sub-populations (~0.35 m/s) are inhibited by IR light (arrow). **(Trace 38)** CAP after IR application for 13 seconds. All components of the CAP are inhibited (arrows). **(Trace 48)** CAP after removal of IR light; all CAP components are present, indicating reversibility. **(a - Right)** Contour plot of CAP traces (electrical stimulation frequency, 2 Hz) illustrating progressive preferential block of slow components during IR application (red vertical bar; on, trace 13; off, trace 44). Conduction velocity (m/s) is plotted against trace number. A color bar indicates the voltages of the traces. Note the sequential inhibition of the slowest conduction velocity components before the intermediate conduction velocity, which in turn are inhibited before the fastest conduction velocity components. **(b)** Plots of medians (dark line) surrounded by the first quartile (upper dashed line) and third quartile (lower dashed lines) for successive groups of 6 recordings with conduction velocity between 1.24 m/s and 0.51 m/s. Note the reduction in median value when all components of the CAP are blocked. **(c)** Plots of medians (dark line) surrounded by the first quartile (upper dashed line) and third quartile (lower dashed lines) for successive groups of 6 recordings with conduction velocity between 0.51 and 0.18 m/s. Note the reduction in the median value earlier than the reduction observed in the faster components of the CAP. Using a chi-squared test, slow-velocity components (0.51 – 0.18 m/s) showed statistically significant reductions in RAUC when compared to fast-velocity components (1.24 - 0.51 m/s). Slow-velocity components in a third experiment also showed statistically significant reductions in RAUC when compared to fast-velocity components (data not shown).


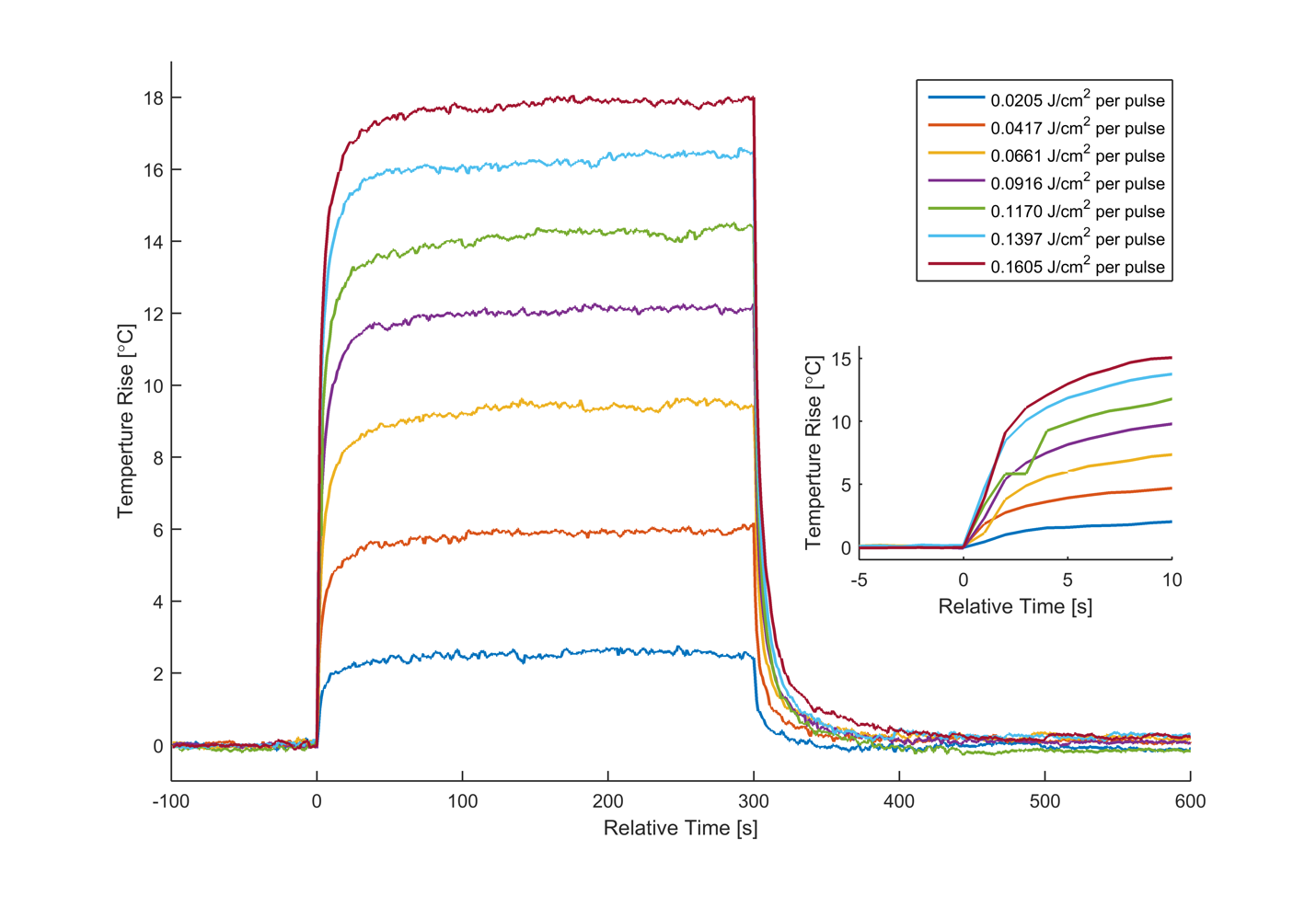
**Figure S6: Temperature increase using parameters inhibiting axons in *Aplysia californica.*** See Methods for the detailed description of how measurements were performed. Application of IR light (wavelength: 1860 nm, optical fiber diameter: 600 µm) over a range of radiant exposures (see legend for values) caused rapid temperature increases (inset shows changes during the first 10 seconds of IR application).


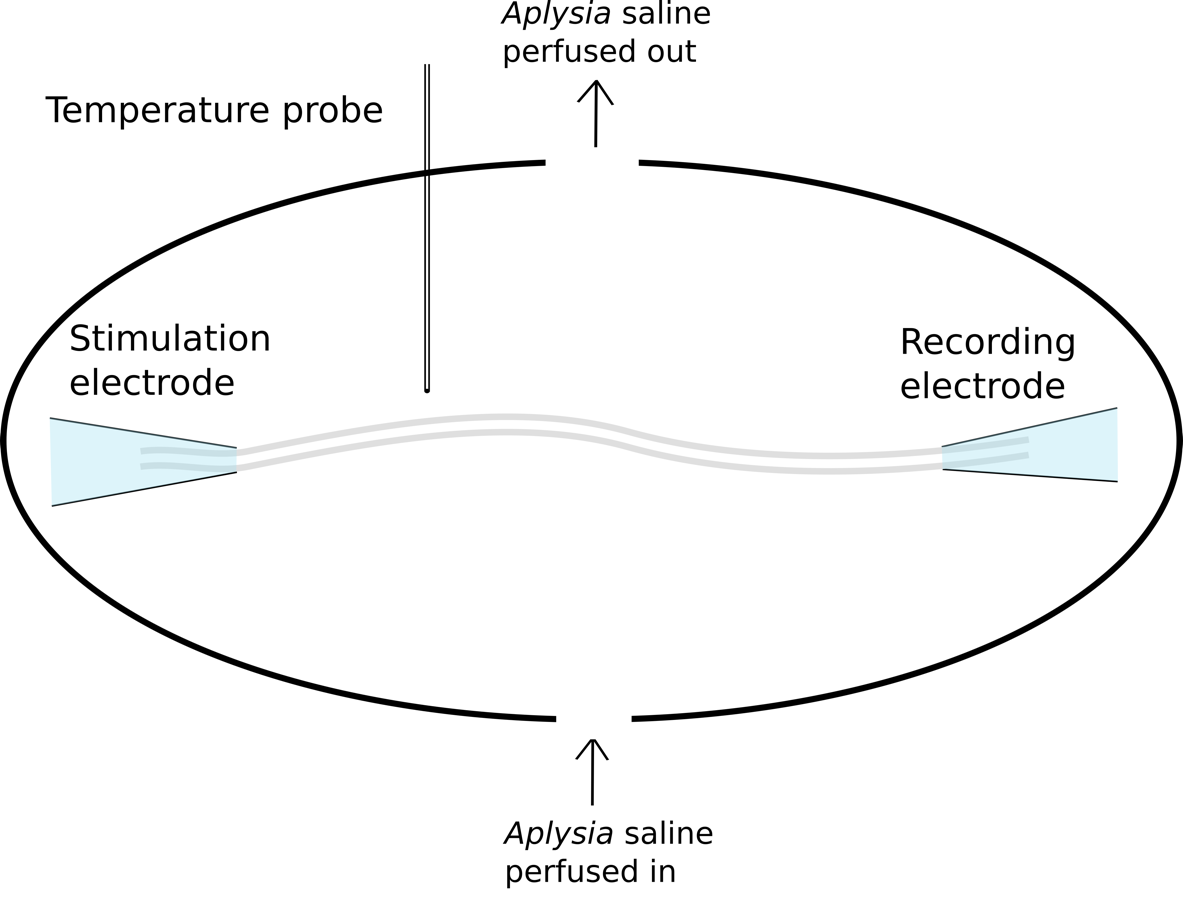


**Figure S7: Experimental setup for bath heating inhibition in the pleural-abdominal connective of *Aplysia californica*.** A stimulation suction electrode was placed on one end of the nerve (left) and a recording suction electrode was placed on the other end of the nerve (right). *Aplysia* saline at varying temperatures was perfused into the dish. A temperature probe was placed close to the nerve to monitor saline temperature.


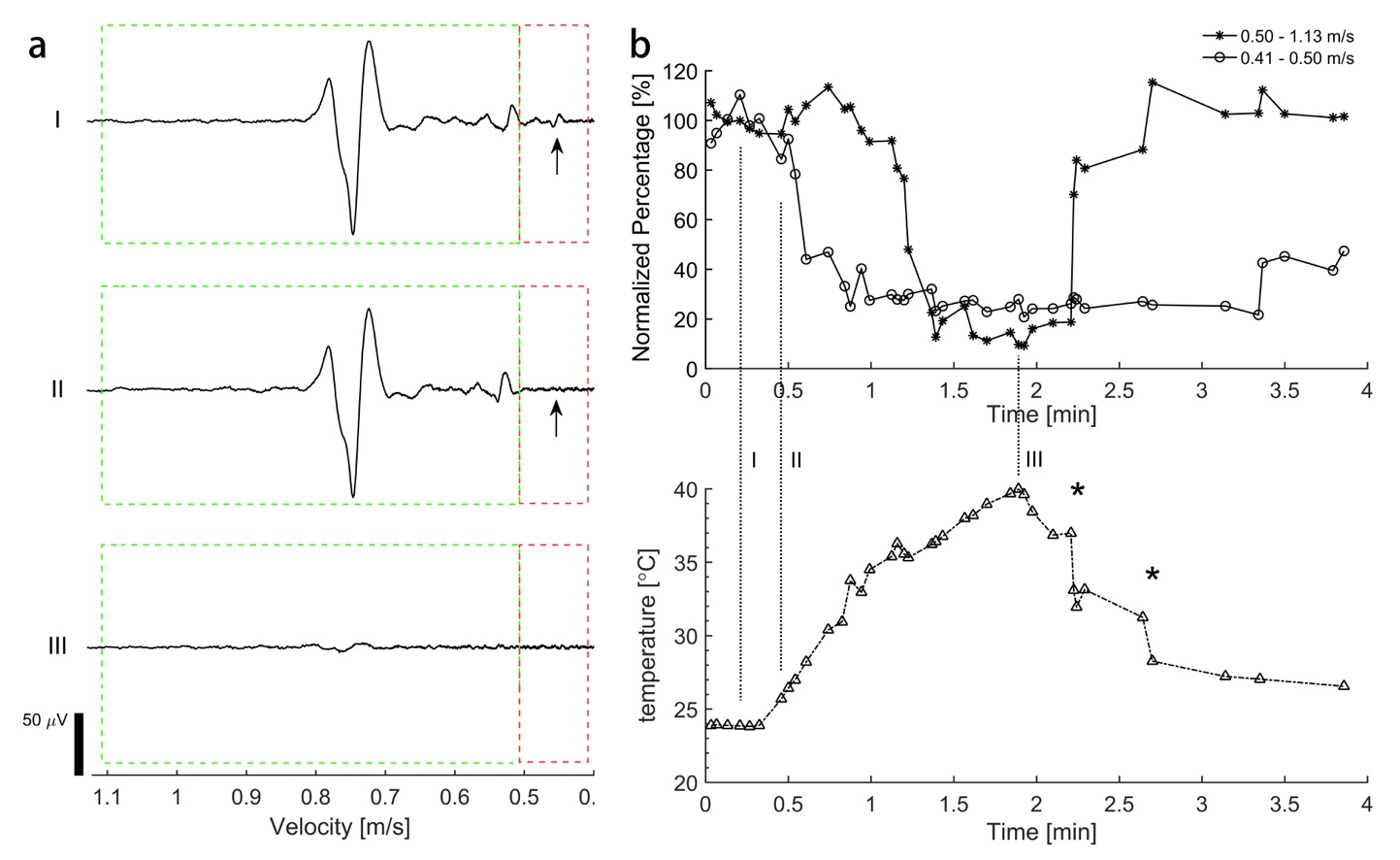


**Figure S8: Whole nerve heating produces preferential inhibition of slower-conducting components of *Aplysia californica* compound action potential.** **(a)** Slower-conducting components of the CAP are blocked earlier when the whole nerve is heated. **(I)** CAP at 23.8°C. The green box delineates the components of the CAP with a conduction velocity 0.50-1.13 m/s. The red box delineates the components of the CAP with a conduction velocity 0.41-0.50 m/s. **(II)** CAP at 25.7°C. The slowest component of the CAP is blocked (arrow). **(III)** CAP at 40°C. All components of the CAP are blocked. **(b)** Normalized percentage of the rectified area under the curve for the two CAP sub-populations at corresponding temperatures. The slowest sub-population (0.41-0.50 m/s) is clearly affected at lower temperatures (starting at ~26°C) than the faster sub-populations (0.50-1.13 m/s) that require higher temperatures (starting at ~35°C) to be affected. Cool saline was used to lower the bath temperature (stars), so that the nerve’s health after warming could be assessed.

**Section 4. Inhibition of small, slow-conducting fibers in whole nerves of the**

**shrew**

**
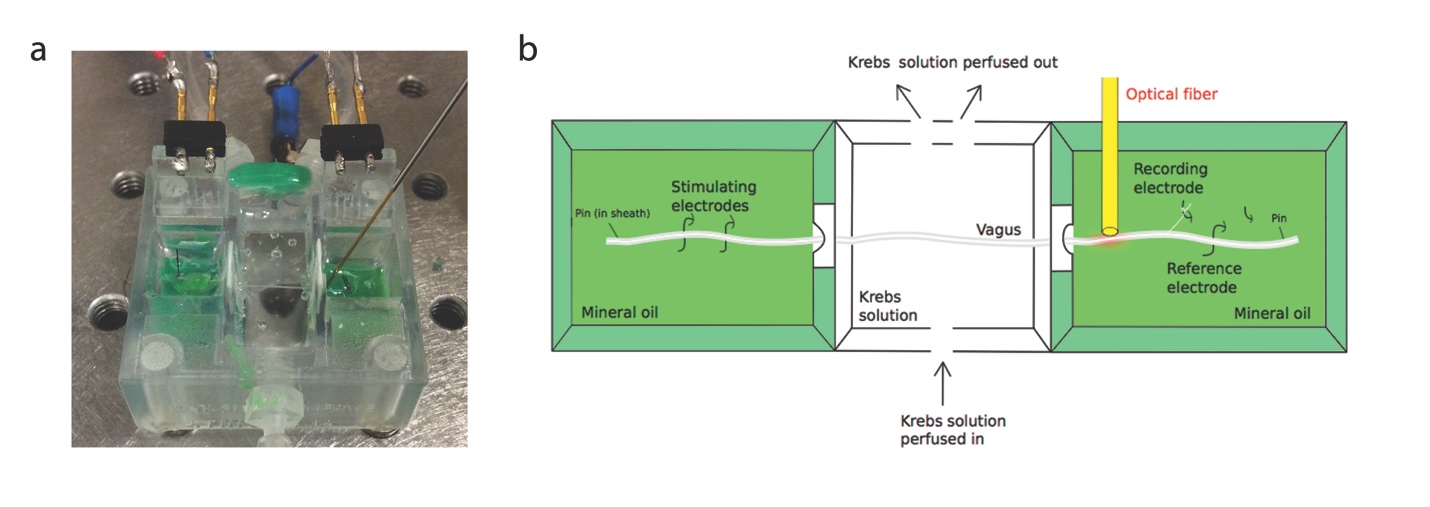
Figure S9: *in vitro* experimental setup for optical inhibition in the vagus nerve of *Suncus murinus*.** **(a)** Photograph of custom 3-D printed three-chambered platform (~3.6 X 3.4 X 1.4 cm) used to perform *in vitro* experiments (http://3dprint.nih.gov/discover/3dpx-003133). **(b)** Schematic of experimental setup. The vagus nerve was stretched across three chambers. The stimulating and recording chambers (green) were filled with mineral oil to reduce current spread, and the central chamber (white) was perfused with Krebs solution. Krebs solution was heated to ensure that it would be 37 ^o^C when it entered the middle chamber. In the stimulating chamber, the nerve was draped over electrodes. In the recording chamber, the nerve was locally desheathed and individual fiber bundles were dissected and wrapped around the recording electrode. The optical fiber (400 µm diameter, 1860 nm wavelength) was positioned perpendicularly to the nerve proximal to the recording electrode. The temperature at the site of laser application was close to body temperature.


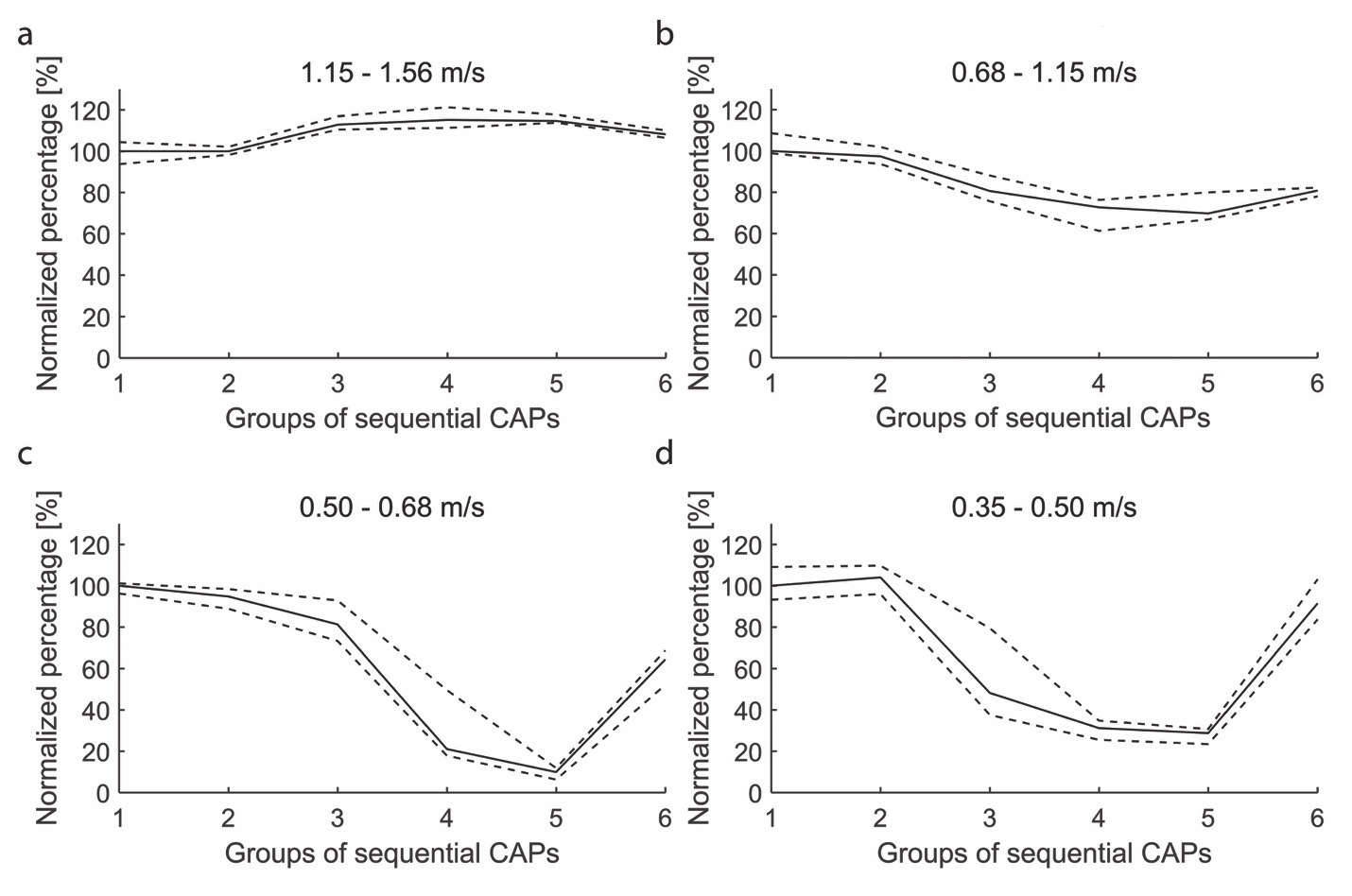


**Figure S10: Quantifying change of the rectified area under the curve for CAP components in *Suncus murinus*** **(a) – (d)** Plots of medians (dark lines) surrounded by the first quartile (upper dashed lines) and third quartile (lower dashed lines) for successive groups of 10 recordings in the different conduction velocity ranges (a: 1.56 - 1.15 m/s, b: 1.15 - 0.68 m/s, c: 0.68 - 0.50 m/s, d: 0.50 - 0.35 m/s) of the CAP. These results quantify the data shown in Figure 4. The experiment was repeated three times. Using Cochran-Mantel-Haenszel tests, slow-velocity components (c and d) showed statistically significant reductions in RAUC when compared to fast-velocity components (a and b). The slowest fibers are inhibited before the other conduction velocity components.


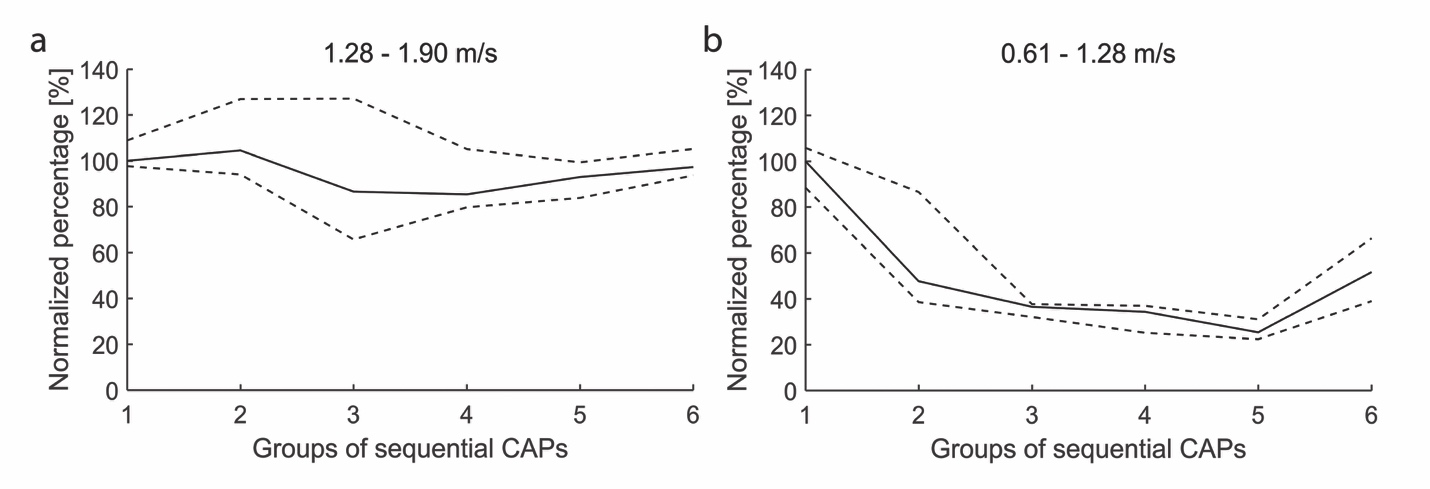


**Figure S11:** **Quantifying change of the rectified area under the curve for CAP components in *Suncus murinus*.** Results from a second animal compared to Figure S8. (a) – (b) Plots of medians (dark lines) surrounded by the first quartile (upper dashed lines) and third quartile (lower dashed lines) for successive groups of 10 recordings in the different conduction velocity ranges (a: 1.90 - 1.28 m/s, b: 1.28 - 0.61 m/s) of the CAP. Using Cochran-Mantel-Haenszel tests, slow-velocity components (b) showed statistically significant reductions in RAUC when compared to fast-velocity components (a). The slow-velocity components in a third animal also showed statistically significant reductions in RAUC when compared to fast-velocity components (data not shown).


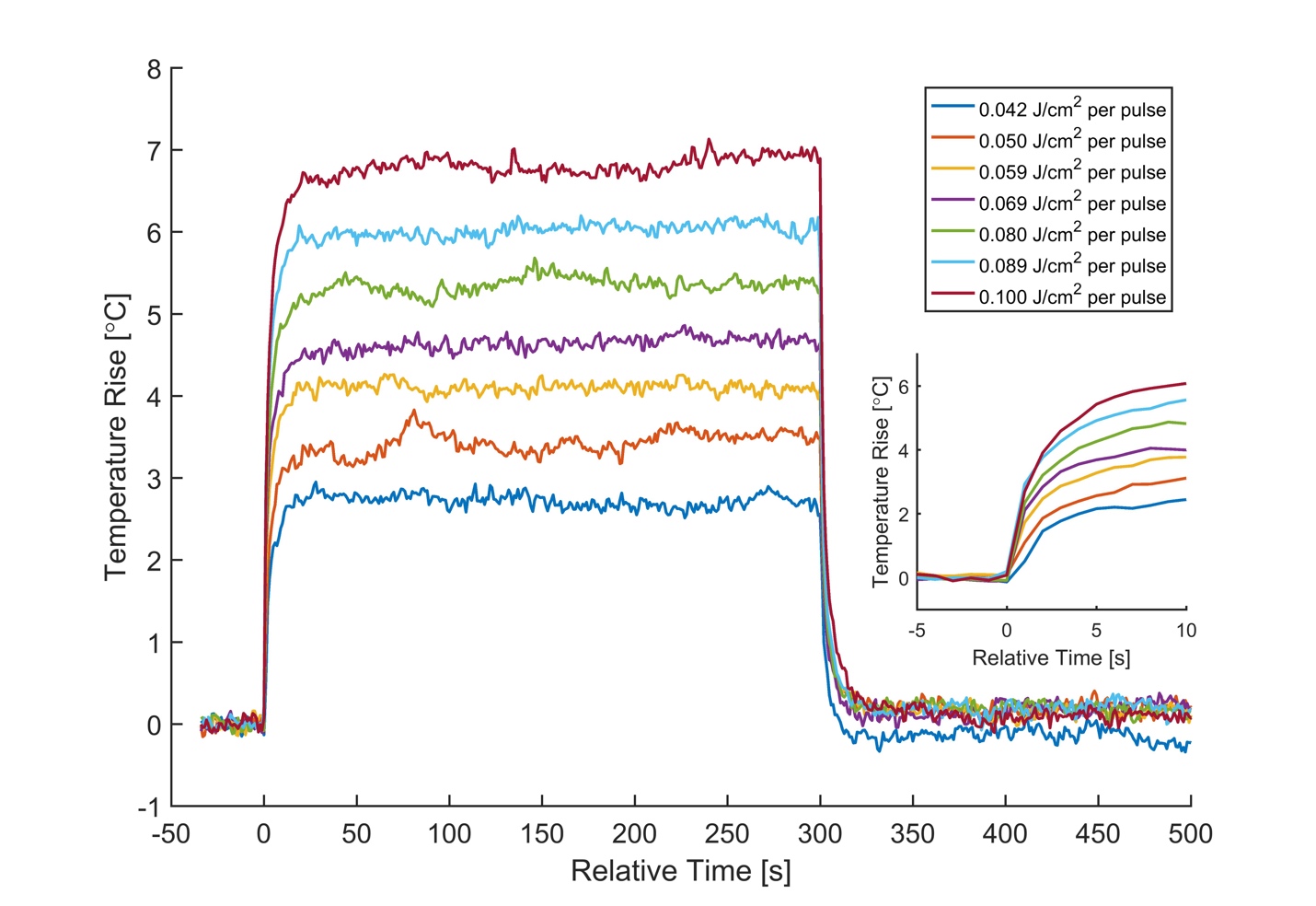


**Figure S12: Temperature increase using parameters inhibiting axons in *Suncus murinus.*** See Methods for the detailed description of how measurements were performed. Application of IR light (wavelength: 1860 nm, optical fiber diameter: 400 µm) over a range of radiant exposures (see legend for values) caused rapid temperature increases (inset shows changes during the first 10 seconds of IR application).

**
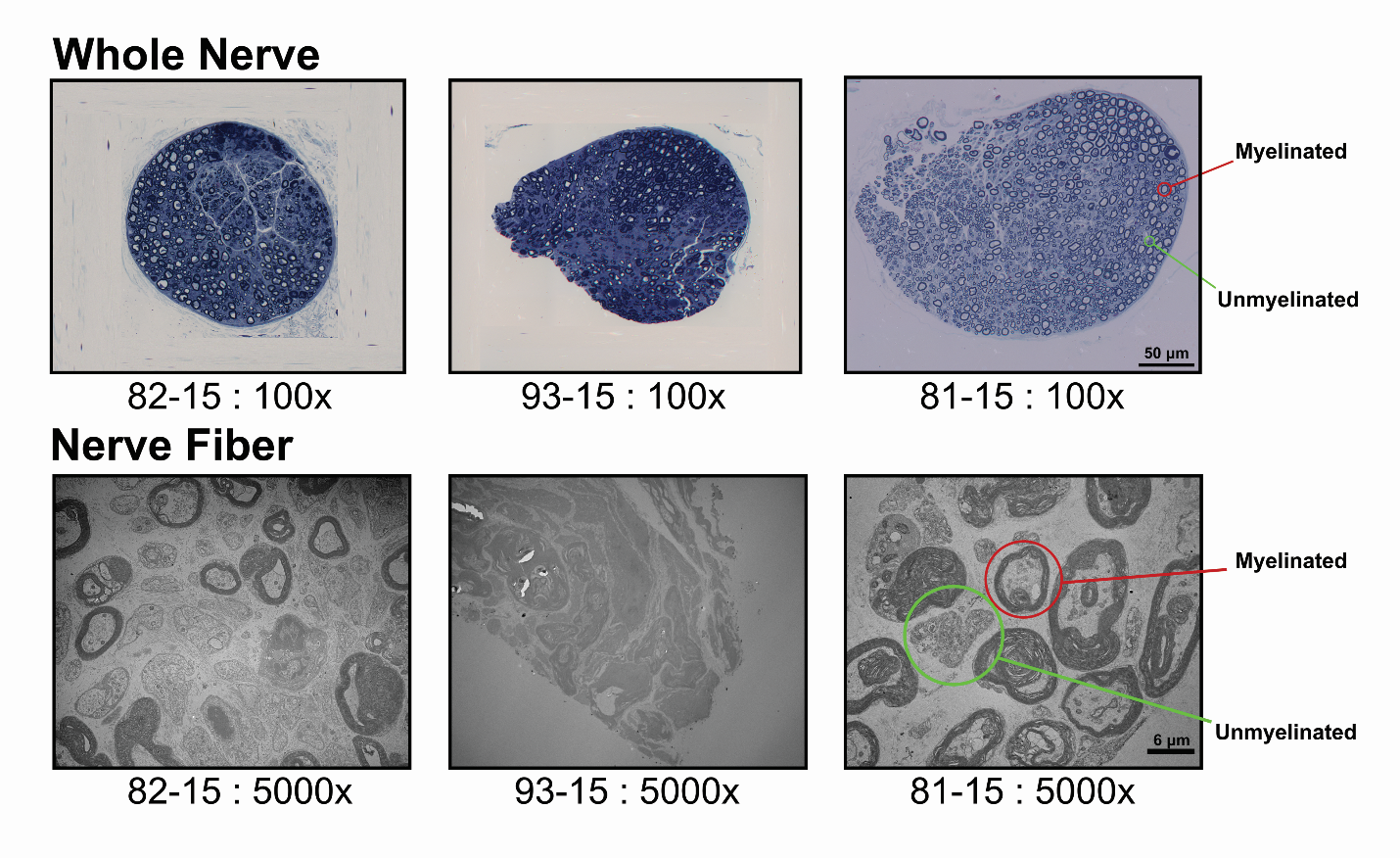
**

**Figure S13: Histology of the cervical vagus of the musk shrew.** **Top**: Toluidine blue staining of transverse sections of the whole nerve from the three animals used in the physiology experiments. **Bottom**: Transmission electron microscopy (TEM) images from the three recorded nerve fibers (bundles). Animal numbers and magnification levels are indicated below each image.
